# National Institutes of Health–Defined Chronic Graft-vs.-Host Disease in Pediatric Hematopoietic Stem Cell Transplantation Patients Correlates With Parameters of Long-Term Immune Reconstitution

**DOI:** 10.3389/fimmu.2019.01879

**Published:** 2019-08-27

**Authors:** Anita Lawitschka, Ece Dila Gueclue, Angela Januszko, Ulrike Körmöczi, Arno Rottal, Gerhard Fritsch, Dorothea Bauer, Christina Peters, Hildegard T. Greinix, Winfried F. Pickl, Zoya Kuzmina

**Affiliations:** ^1^Children's Cancer Research Institute, St. Anna Children's Hospital, Medical University of Vienna, Vienna, Austria; ^2^Center for Pathophysiology, Infectiology and Immunology, Institute of Immunology, Medical University of Vienna, Vienna, Austria; ^3^Division of Hematology, Medical University of Graz, Graz, Austria

**Keywords:** pediatric hematopoietic stem cell transplantation, biomarker, B-cells, immune reconstitution, chronic graft-vs.-host disease

## Abstract

Recent data revealed the importance of immune reconstitution (IR) for the evaluation of possible biomarkers in National Institutes of Health (NIH)–defined chronic graft-vs.-host disease (cGVHD) and its clinical aspects. In this large pediatric study (*n* = 146), we have analyzed whether cellular and humoral parameters of IR in the long-term follow-up (FU) with a special emphasis on B-cell reconstitution correlate with NIH-defined cGVHD criteria. HYPOTHESIS: we were especially interested in whether meaningful cGVHD biomarkers could be defined in a large pediatric cohort. We here demonstrate for the first time in a highly homogenous pediatric patient cohort that both cGVHD (*n* = 38) and its activity were associated with the perturbation of the B-cell compartment, including low frequencies of CD19^+^CD27^+^ memory B-cells and increased frequencies of circulating CD19^+^CD21^low^ B-cells, a well-known hyperactivated B-cell subset frequently found elevated in chronic infection and autoimmunity. Notably, resolution of cGVHD correlated with expansion of CD19^+^CD27^+^ memory B-cells and normalization of CD19^+^CD21^low^ B-cell frequencies. Moreover, we found that the severity of cGVHD had an impact on parameters of IR and that severe cGVHD was associated with increased CD19^+^CD21^low^ B-cell frequencies. When comparing the clinical characteristics of the active and non-active cGVHD patients (in detail at time of analyses), we found a correlation between activity and a higher overall severity of cGVHD, which means that in the active cGVHD patient group were more patients with a higher disease burden of cGVHD—despite similar risk profiles for cGVHD. Our data also provide solid evidence that the time point of analysis regarding both hematopoietic stem cell transplantation (HSCT) FU and cGVHD disease activity may be of critical importance for the detailed investigation of pediatric cohorts. Finally, we have proven that the differences in risk factors and patterns of IR, with cGVHD as its main confounding factor, between malignant and non-malignant diseases, are important to be considered in future studies aiming at identification of novel biomarkers for cGVHD.

## Introduction

Chronic graft-vs.-host disease (cGVHD) is a multisystem immune disorder occurring in 40–70% of patients after allogeneic hematopoietic stem cell transplantation (HSCT). It is the leading cause of long-term non-relapse mortality (NRM) mainly associated with delayed immune reconstitution (IR) and infections (1). In pediatric patients, the incidence is lower (5–30%), but the sequelae may be more detrimental since they occur in a growing organism (2). However, GVHD can also be regarded as being “protective,” since patients who experienced cGVHD have lower rates of recurrence of their underlying malignant disease (3). One important aspect regarding pediatric patients undergoing HSCT is the fact that up to 50% of transplantations are being performed for non-malignant underlying diseases. In all such cases, no benefit from the graft-vs.-malignancy effect due to cGVHD can be deduced.

The kinetics of IR seem to be disturbed in cGVHD patients (4), while other common HSCT complications such as infections, relapses or secondary malignancies are also associated with the failure of proper IR (5–7). Data on pediatric IR mainly cover the first year after transplantation (8) and compare IR and outcome data by various graft sources and the use of serotherapy (9). Studies on pediatric IR regarding the various underlying malignant and non-malignant diseases are limited. Moreover, since pediatric IR is dependent on age-related physiological aspects (e.g., thymic function, hormones), there is a need—currently unmet—of harmonized pediatric studies, covering the dynamics over time (9).

Recently, the National Institutes of Health (NIH) cGVHD Consensus Group made a number of recommendations regarding criteria for diagnosis, staging, response evaluation, and biomarkers for cGVHD (10, 11). At present, meaningful cGVHD biomarkers are scarce, especially in larger pediatric cohorts (12). As recently outlined by Hilgendorf et al. (13), pediatric data seem limited by either evaluating CD19^+^ cells alone (9) or patient subgroups without the influence of underlying diseases or age (14).

In 2004, we started an observational study with the aim to implement the NIH criteria into daily clinical routine. In parallel, we conducted a prospective, non-interventional study on IR with the aim to identify possible biomarkers for pediatric cGVHD and implemented those parameters into our routine of post-transplant care. Herein, we merged the data of these two studies and investigated associations between NIH-defined cGVHD and pediatric aspects, such as age and underlying malignant or non-malignant disease, covering not only the influence of myeloablative conditioning (MAC) vs. reduced-intensity conditioning (RIC) regimens but also the influence of pre-HSCT treatment and genetic disposition. Since IR might differ between patients with malignant and non-malignant diseases, we investigated whether patterns of cGVHD are different and correlated them with cellular and humoral parameters of IR with a special focus on B-cell perturbations, which have been described to be of importance in adult cGVHD patients recently (15, 16). We have chosen the long-term follow-up (FU) to minimize effects of engraftment kinetics and to enhance the number of evaluations of cGVHD patients.

Chronic hyperactivation of the immune system, as observed in cGVHD, might generate an inflammatory milieu advantageous for breaking B-cell tolerance and inhibiting negative selection and maturation of B-cells (17–19). Accordingly, levels of hyperactivated, exhausted CD19^+^CD21^low^CD27^−^ tissue-like memory B-cells and reduced B-cell receptor (BCR)–induced immunoglobulin-secreting capacity, as observed frequently in individuals with hepatitis C infection (20), Sjogren's syndrome (21, 22), and HIV (23, 24), might become apparent also in pediatric patients suffering from cGVHD. Of relevance, distortions involving several B-cellular subsets/maturational stages such as low proportions of CD19^+^CD27^+^ memory B-cells and increases in CD19^+^CD21^−^ B-cells have been observed as risk factors/biomarkers in adult cGVHD patients in previous studies (15, 16, 25–27); however, no such investigations could be performed in large pediatric patient groups due to the lack of homogeneous study collectives. Because of our previous findings in adult cGVHD patients, we were especially interested in whether the B-cellular compartment is dynamically formed and influenced after HSCT in the presence or absence of cGVHD and whether its actual configuration at given time points after HSCT would correlate with the activity of cGVHD.

Consequently, we followed a large cohort of children (*n* = 146) who underwent HSCT for various reasons and during different stages of childhood development. Both the interval from HSCT and the activity of NIH-defined cGVHD at the time of analyses were considered, as we aimed for clinical meaningfulness and reflection upon the reconstitution process, making this study one of the largest pediatric studies on long-term IR and NIH-defined cGVHD described so far (28).

## Methods

### Patients

Between February 2004 and March 2012, 146 pediatric patients (defined as number = *n*) who were a minimum of 100 days after HSCT or suffering from ongoing cGVHD were enrolled into this study at the HSCT Outpatient Clinic of St. Anna Children's Hospital. Of these, 35 patients participated in a prospective, non-interventional study on IR; results were then merged with IR parameters routinely performed during aftercare. Analyses (defined as analyses = a) were grouped according to (i) the interval from HSCT, i.e., early FU (before day +365) and late FU (after day +365), and (ii) cGVHD activity, i.e., *no* (never) cGVHD or *active* and *resolved* cGVHD. Supplemental Tables 1, 2 include general patient characteristics as well as age at time point of analyses and interval from HSCT to analyses.

Inclusion criteria covered first HSCT, lack of life-threatening infections, survival expectation more than 5 months, and complete remission of the underlying disease. Exclusion criteria were incomplete engraftment and prior treatment with rituximab. Written informed consent in accordance with the Declaration of Helsinki and the institutional review board of the Medical University of Vienna and St. Anna Children's Hospital had been obtained. Laboratory and clinical evaluations were done after day +100 every 3–4 months in the first year, every 6 months in the second year, once a year afterwards, and when clinically indicated.

Standard GVHD prophylaxes were applied according to international and institutional protocols. Patients were monitored for cytomegalovirus, Epstein–Barr virus, and adenovirus reactivation with polymerase chain reaction assays, and received antimicrobial and antifungal prophylaxis according to institutional guidelines. Chimerism was tested on sorted leukocyte subsets in peripheral blood (PB) by standardized variable number tandem repeat (VNTR) analysis until persistent full donor or stable mixed chimerism was reached. Acute GVHD (aGVHD) was scored using the modified *Glucksberg* criteria (29). NIH consensus criteria were applied for diagnosis and staging of cGVHD patients after 2005 and re-evaluated in all other patients (10).

### Samples

We analyzed numbers and distribution of leukocytes and major T- and B-cell subsets in PB and measured serum immunoglobulin (Ig) levels at consecutive time points after HSCT. The following assessments were done longitudinally: leukocytes, lymphocytes, monocytes, granulocytes, total IgG and IgG subclasses 1–4, IgM, IgA, IgE, T-cell subpopulations (CD3^+^, CD4^+^, CD8^+^, ratio CD4^+^/CD8^+^), natural killer (NK) cells (CD3^−^CD56^+^CD16^+^), and B-cell subsets (CD19^+^, CD19^+^CD27^+^, CD19^+^CD27^+^IgD^+^ non-class-switched and CD19^+^CD27^+^IgD^−^ class-switched memory B-cells, CD19^+^CD21^low^ B-cells). Optimal concentrations of directly conjugated monoclonal antibodies (Supplemental Table 3) were added to 50 μl of patients' whole blood and incubated at room temperature for 20 minutes. ADG lysis solution (An der Grub, Vienna, Austria) was used to remove red blood cells according to the manufacturer's recommendations followed by acquisition of 5 × 10^3^ cells in the lymphogate for leukocyte subpopulations and 4–8 × 10^3^ CD19^+^ B-cells for B-cell subset analysis as described (15). Reference serum levels of IgG/M/A/E were quantified by nephelometry (BNII, Dade Behring, Marburg, Germany). Supplemental Table 4 shows reference values for Ig and IgG-subclasses for the different age groups.

### Statistical Analyses

Fisher's exact test was used to examine the significance of the association between two variables. Statistical pair-wise comparisons of cellular subsets within each patient group were made using the unpaired Student's *t*-test. For univariate analyses, different subpopulations and clinical cGVHD details at the time of analyses throughout the long-term FU were selected. Pearson's correlation and logistic regression analyses for factors impacting cellular and humoral parameters were performed. Area under the curve (AUC) and corresponding 95% confidence intervals (CIs) were computed non-parametrically. Covariates with a *P*-value <0.05 were entered into the multivariate analyses. The data were calculated using SPSS 20.0 (IBM Company, Chicago, IL).

## Results

The 146 pediatric study patients (defined as *n* = number of patients) with a median FU of 8.6 years (range, 0.4–19.3 years) underwent consecutive measurements, and overall, 659 specimens (defined as a = number of analyses) were collected (flow diagram). Acute GVHD was diagnosed in 93 patients (64%); after NIH-defined re-evaluation, 7 patients (8%) with late aGVHD were excluded. Chronic GVHD was diagnosed in 38 patients (26%) at a median onset of 6 months (range, 2.5–48 months) after HSCT, with manifestations of classic cGVHD in 25 (66%) and overlap syndrome in 13 children (34%). Risk factor evaluation included a history of aGVHD in the majority of patients (87%) and thrombocytopenia (i.e., platelet counts <100 G/L) in 10 cases (26%) at onset of cGVHD. In the subgroup of cGVHD patients with malignant underlying diseases (*n* = 29), which we will focus on as described later, the median onset interval and the percentage of classic chronic and overlap cGVHD were similar. The onset type of cGVHD was *quiescent* in 21 (55%), *progressive* in 13 (34%), and *de novo* in 4 patients (10%).

When comparing patient and transplant characteristics between the cGVHD and no-cGVHD group, no significant differences were observed regarding age, gender, underlying diseases, conditioning regimen including total body irradiation (TBI), donor and stem cell sources, median number of CD34^+^ cells/kg transplanted, survival, and FU. In contrast, the cGVHD group received significantly less antithymocyte globulin (ATG) accompanied by a higher incidence and greater severity of aGVHD (S1).

Age and time interval since HSCT—both of which may be crucial for IR—were evaluated at all study time points. Notably, patients of the cGVHD group were significantly older (median age 13.4 vs. 12 years, *p* = 0.02) with longer intervals from HSCT when compared to the no-cGVHD group (S2).

### Significant Differences Regarding Risk Factors and Incidence of GVHD When Comparing Malignant and Non-malignant Underlying Diseases

Next, we performed a sub-analysis of patients and transplant characteristics according to the malignant and non-malignant underlying diseases (Table 1), and the following significant differences were observed: median age (11.1 vs. 5.6 years), RIC (18% vs. 94%), TBI conditioning (66% vs. 4%), TCD (7% vs. 30%), MMRD (mostly haploidentical, 3 vs. 16%), and stem cell source (BM 78% vs. 56%; peripheral blood stem cells (PBSC) 22% vs. 44%). The wider application of PBSCs in non-malignant HSCT is reflected by the difference in CD34^+^ cells/kg transplanted. In addition, GVHD prophylaxis significantly differed between the two groups: CsA + MTX (44% vs. 8%), CsA + MMF (11% vs. 48%), and ATG (64% vs. 88%). Notably, the incidence of both aGVHD and cGVHD was higher in patients with malignant disease (significant for aGVHD). Characteristics of cGVHD like overall severity, organ scoring, onset time, and duration of FU did not differ significantly.

**Table 1:** Patient characteristics regarding underlying disease (malignant vs. non-malignant).

**Number of patients**	**Malignant *n* = 91 (%)**	**Non-malignant *n* = 48 (%)**	***p* (*x*^**2**^ test/test)**
Median age at HSCT in years (range)	11.1 (1–23.8)	5.6 (0.1–24.8)	<0.0001
Male	63 (69)	28 (58)	n.s.
Female	28 (31)	20 (42)	
**Conditioning regimen**			
MAC	75 (82)	3 (6)	0.0001
RIC	16 (18)	45 (94)	
TBI containing	60 (66)	2 (4)	0.0001
TBI+MAC	57 (95)	0	0.0053
TCD *ex vivo*	6 (7)	15 (30)	0.0003
**Stem cell donors**			
MRD	34 (37)	17 (35)	n.s.
MUD	52 (57)	21 (44)	n.s.
MMRD	3 (3)	8 (16)	0.0081
MMUD	2 (2)	2 (4)	n.s.
**Stem cell source**			
Bone marrow	71 (78)	27 (56)	0.0075
PBSCs	20 (22)	21 (44)	
Median number of CD34^+^ cells × 10^6^/kg (range)	6.0 (0.4–62)	10.7 (0.24–40)	<0.0001
**Post-transplant immunosuppressive prophylaxis**			
No GVHD prophylaxis	2 (2)	3 (6)	n.s.
CsA only	37 (41)	13 (27)	n.s.
CsA+MTX	40 (44)	4 (8)	0.0001
CsA+MMF	10 (11)	23 (48)	0.0001
CsA+MTX+MMF	1 (1)	3 (6)	n.s.
CsA+FK506	1 (1)	0	n.s.
+ATG^*^	61 (64)	44 (88)	0.0018
**Acute GVHD**	66 (73)	19 (40)	0.0005
Grade 0	25 (27)	29 (60)	
Grade 0–I	63 (69)	43 (90)	0.025
Grade II–IV	28 (42)	5 (24)	0.0825
Chronic GVHD	29 (30)	9 (18)	n.s.
Median time from HSCT to onset of cGVHD in months (range)	6 (3–18)	10 (3–50)	n.s.

* *ATG was given additionally to other conditioning medications*.

### Immune Reconstitution in a Homogenous Cohort Without the Effect of cGVHD: Several Cellular and Humoral Parameters Still Reconstitute After Day +365 Post HSCT, but Frequencies of Circulating CD19^+^ B-cells Decrease

**Figure 1:**
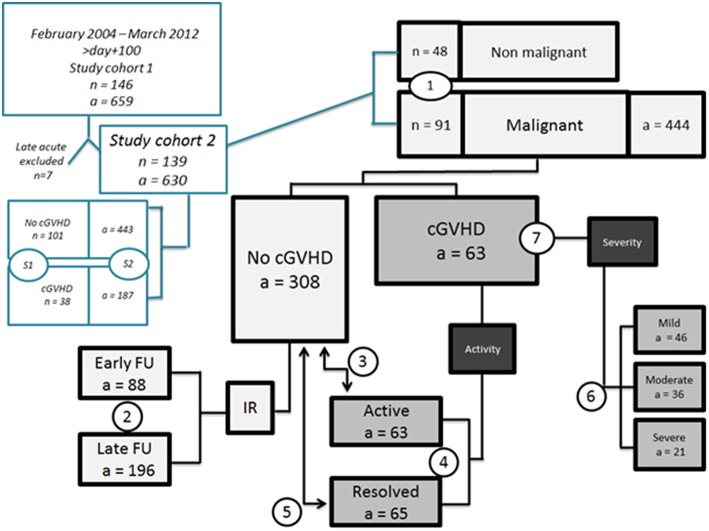
Flow diagram. S1 General patient characteristics of study cohort (after exclusion of late acute GVHD); S2 age at time point of analyses and interval from HSCT to analyses; (1) patient characteristics regarding underlying diseases (comparison between malignant and non-malignant cohorts); (2) IR parameters in malignant diseases in no cGVHD cohort early (*n* = 47, a = 88) vs. late FU (*n* = 56, a = 197); (3) comparison between active (*n* = 29, a = 63) vs. no cGVHD (*n* = 67, a = 308) malignant only; (4) comparison between active (*n* = 29, a = 63) vs. resolved (*n* = 20, a = 65) cGVHD; malignant only; (5) comparison of resolved vs. no cGVHD, malignant only; (6) impact of NIH overall severity on IR parameters, malignant only; (7) clinical cGVHD characteristics of patients with malignant diseases. a, number of analyses. n, number of patients.

Since we observed significant differences between malignant and non-malignant diseases regarding factors that may influence IR, we focused our subsequent evaluations on patients with malignant underlying diseases as HSCT indication (Figure 1).

Concerning the kinetics of IR during long-term FU, we compared “early” (before day +365, *n* = 88 analyses) to “late” (after day +365, *n* = 196 analyses) time points only in patients without any cGVHD (Table 2). Notably, we found significantly lower lymphocyte (1,885.7 vs. 2,323.5 × 10^3^/ml, *p* = 0.002) and monocyte (420.1 vs. 471.1 × 10^3^/ml, *p* = 0.043) numbers during early FU. Analyses of T-lymphocyte subpopulations revealed significantly lower overall CD3^+^ T-cells (1,122.9 vs. 1,521.9 × 10^3^/ml, *p* < 0.0001) and CD4^+^ T-cells (393.3 vs. 716.8 × 10^3^/ml, *p* < 0.0001) and, due to the CD4^+^ deficiency, a distorted CD4/CD8 ratio (0.867 vs. 1.21, *p* < 0.0001) when comparing early with late FU, respectively. Furthermore, patients during early FU presented with lower IgG levels (975.7 vs. 1,091.5 mg/dl, *p* = 0.007), which was mostly due to the reduction of the IgG1 subclass; the same applied to IgA (98.4 vs. 149.5 mg/dl, *p* < 0.0001) and IgE (64.4 vs. 166.7 kU/L, *p* = 0.005) when compared to late FU.

**Table 2:** Immune reconstitution in patients with malignant diseases and without cGVHD (no cGVHD): comparison of early (day + 100 till + 365) to late (>day + 365) time points after HSCT.

**Parameters**	**Early FU a = 88**	**Late FU a = 197**	***P*-value**
Lymphocytes × 10^3^ cells/ml	1,885.7	2,323.5	0.002
Monocytes × 10^3^ cells/ml	420.1	471.1	0.043
IgG mg/dl	975.7	1,091.5	0.007
IgA mg/dl	98.4	149.5	<0.001
IgE kU/L	64.4	166.7	0.005
IgG1 mg/dl	717.2	804.1	0.050
CD3^+^ T-cells × 10^3^ cells/ml	1,122.9	1,521.9	<0.001
CD4^+^ T helper cells × 10^3^ cells/ml	393.3	716.8	<0.0001
CD4/CD8 ratio	0.867	1.21	<0.0001
CD19^+^CD27^+^ %	12.0	17.9	0.008
CD19^+^CD27^+^ × 10^3^ cells/ml	34.8	74.7	<0.0001
CD19^+^CD27^+^IgD^+^ × 10^6^ cells/ml	25.2	46.9	0.026
CD19^+^CD27^+^IgD^−^ %	3.2	7.0	<0.0001
CD19^+^CD27^+^IgD^−^× 10^3^ cells/ml	10.5	27.8	<0.0001
CD19^+^CD21^low^ %	11.1	7.29	0.006
Ratio CD19^+^CD27^+^IgD^+^/IgD^−^	4.9	2.1	0.001
Ratio CD19^+^CD21^low^/CD19^+^CD27^+^	2.0	0.6	<0.0001

*Mean values are depicted, and only significant values are shown*.

*a, number of analyses; FU, follow-up*.

**Figure 2:**
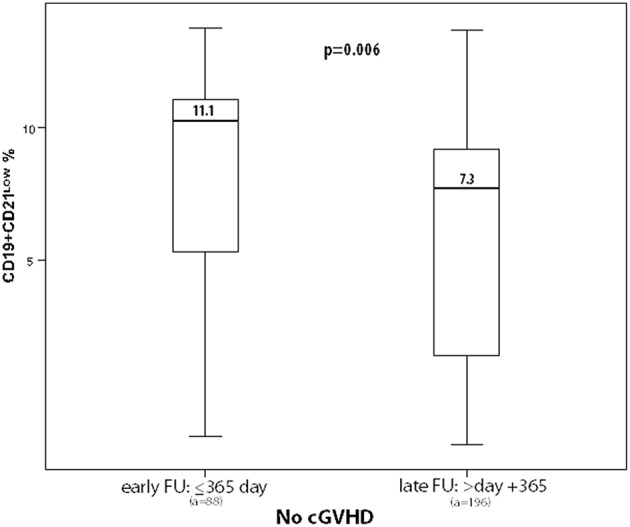
Box plots depict frequencies of CD19^+^CD21^low^ B-cells during long-term follow-up in malignant underlying diseases only, without the effect of cGVHD (a, number of analyses).

While CD19^+^ B-cells did not differ, the proportion of memory CD19^+^CD27^+^ B-cells was diminished during early FU (12% vs. 17.9%, *p* = 0.008). Moreover, absolute numbers of both class-switched and non-class-switched memory (CD19^+^CD27^+^ IgD^−^ and CD19^+^CD27^+^ IgD^+^) B-cells were significantly decreased during early compared to late FU, with 10.5 vs. 27.8 × 10^3^/ml (*p* < 0.0001) and 25.2 vs. 46.9 × 10^3^/ml (*p* = 0.026), respectively. Accordingly, early FU showed significantly higher frequencies of CD19^+^CD21^low^ B-cells when compared to late FU (11.1% vs. 7.29%, *p* = 0.006, Figure 2). No further changes in reconstitution profiles were detected.

### Activity of cGVHD Is Associated With Low Frequencies of CD19^+^CD27^+^ Memory B-cells and Increased Frequencies of CD19^+^CD21^low^ B-cells

For a precise assessment of cGVHD activity, we differentiated at time of analysis between (i) active cGVHD and (ii) resolved cGVHD using the no-cGVHD group as a control group (Tables 3–5). Notably, the no-cGVHD category excluded patients with future and resolved cGVHD.

**Table 3:** Parameters of immune reconstitution in patients with malignant underlying diseases: analyses of patients with active cGVHD compared to no cGVHD.

**Parameters**	**Active cGVHD**	**No cGVHD**	***p***
Number of analyses	a = 63	a = 308	
Leukocytes × 10^3^ cells/ml	7,592.4	6,469.9	0.005
Monocytes × 10^3^ cells/ml	599.0	454.3	0.001
Granulocytes × 10^3^ cells/ml	4,812.5	3,738.6	0.001
IgG mg/dl	1,199.5	1,061.9	0.016
IgM mg/dl	146.8	115.5	0.045
IgG1 mg/dl	901.9	777.0	0.038
IgG2 mg/dl	291.4	238.9	0.013
IgG3 mg/dl	74.9	57.3	0.020
CD56^+^ × 10^3^ cells/ml	305.7	258.1	0.027
CD19^+^ %	23.3	19.4	0.008
CD19^+^CD27^+^ %	11.5	16.3	0.018
CD19^+^CD27^+^IgD^−^× 10^3^ cells/ml	13.9	22.3	0.002
CD19^+^CD21^low^ %	12.1	8.2	0.007
CD19^+^CD21^low^ × 10^3^ cells/ml	49.4	27.8	0.002
Ratio CD19^+^CD21^low^/CD19^+^CD27^+^	1.8	1.0	0.009

*Mean values are depicted, and only significant values are shown*.

*a, number of analyses*.

When comparing parameters of the “active” cGVHD to the “no” cGVHD subgroup, disease activity was associated with a significant increase of leukocytes (7,592.4 vs. 6,469.9 × 10^3^/ml), monocytes (599.0 vs. 454.3 × 10^3^/ml), granulocytes (4,812.5 vs. 3,738.6 × 10^3^/ml), and NK cells (305.7 vs. 258.1 × 10^3^/ml, *p* = 0.027, Table 3). IgG levels were within the physiological range in all patients but found to be increased in active when compared to no cGVHD (1,199.5 vs. 1,061.9 mg/dl, *p* = 0.016) and concerning IgG1–3 subclasses. Likewise, elevated IgM levels (146.8 vs. 115.5 mg/dl, *p* = 0.045) were found. The proportion of CD19^+^ B-cells was slightly elevated in active cGVHD (23.3% vs. 19.4%, *p* = 0.008).

However, CD19^+^CD27^+^ memory B-cells were diminished in active cGVHD (11.5% vs. 16.3%, *p* = 0.018; 13.9 vs. 22.3 × 10^3^/ml, *p* = 0.002). In contrast, CD19^+^CD21^low^ B-cells were significantly expanded in active cGVHD (12.1% vs. 8.2%, *p* = 0.007, Figure 2); similarly, the CD19^+^CD21^low^/CD19^+^CD27^+^ B-cell ratio was elevated (1.8 vs. 1.0, *p* = 0.009, Table 3).

### Resolution of cGVHD Correlates With Expansion of CD19^+^CD27^+^ Memory B-cells and Normalization of CD19^+^CD21^low^ B-cell Frequencies

To assess the impact of resolution of cGVHD, data were compared between the “resolved” and the “active” cGVHD subgroup. Of note, we observed a significant trend toward normalization of the B-cell compartment with significant increases in (i) the percentages of CD19^+^CD27^+^ memory B-cells (*p* = 0.005) and of non-class-switched memory B-cells (*p* = 0.030) and (ii) class-switched memory B-cells (absolute count and percentage, *p* < 0.0001) in resolved cGVHD. Moreover, the increased frequencies of CD19^+^CD21^low^ B-cells during active cGVHD significantly decreased from 12.1 to 7.8% (*p* = 0.03), with resolution of cGVHD being accompanied by a significant decrease of the CD19^+^CD21^low^/CD19^+^CD27^+^ ratio (*p* = 0.009, Table 4).

**Table 4:** Parameters of immune reconstitution in patients with malignant underlying diseases: analyses of patients with active cGVHD compared to resolved cGVHD.

**Parameters**	**Active cGVHD**	**Resolved cGVHD**	***P*-value**
Number of analyses	a = 63	a = 65	
IgG2 mg/dl	291.4	233.4	0.023
CD19^+^CD27^+^ %	11.5	19.7	0.005
CD19^+^CD27^+^IgD^+^ %	6.9	12.2	0.030
CD19^+^CD27^+^IgD^−^ %	4.6	7.5	0.006
CD19^+^CD27^+^IgD^−^× 10^3^ cells/ml	13.9	29.9	0.001
CD19^+^CD21^low^ %	12.1	7.8	0.030
Ratio CD19^+^CD21^low^/CD19^+^CD27^+^	1.8	0.9	0.009

*Mean values are depicted, and only significant values are shown*.

*a, number of analyses*.

To determine whether cGVHD resolution would lead to improved and somehow normalized IR, we correlated data of patients with resolved cGVHD with those of the no-cGVHD group (Table 5). Patients with resolved cGVHD still presented with significantly increased leukocyte (7,506.3 vs. 6,469.9 × 10^3^/ml, *p* = 0.006), monocyte (532.7 vs. 454.3 × 10^3^/ml, *p* = 0.004), and granulocyte counts (4,687.3 vs. 3,738.6 × 10^3^/ml, *p* = 0.001). Serum IgG levels (1,177.4 vs. 1,061.9 mg/dl, *p* = 0.015), mainly caused by an increase of the IgG1 (916.4 vs. 777.0 mg/dl, *p* = 0.007) and IgG3 (73.9 vs. 57.3 mg/dl, *p* = 0.004) subclass, were elevated along with increased IgE levels (235.1 vs. 126.6 kU/L, *p* = 0.035). In contrast, IgM levels normalized. Remarkably, class-switched memory B-cells improved significantly. Signs of aberrant B-cell reconstitution, such as increased frequencies of CD19^+^CD21^low^ B-cells along with an increased CD19^+^CD21^low^/CD19^+^CD27^+^ ratio, showed a clear tendency toward normalization and were similar in both groups. These results could be confirmed by *Pearson's* correlations tests, in which cGVHD resolution strongly correlated with the expansion/normalization of CD19^+^CD27^+^ memory B-cells, involving both the class-switched and non-class-switched compartment, and negatively correlated with the CD19^+^CD21^+^/CD19^+^CD27^+^ ratio (data not shown). Moreover, the performed *Spearman* correlation analyses revealed the following significantly distorted parameters during active cGVHD: leukocytes, monocytes, granulocytes, CD56^+^ NK cells, CD19^+^CD21^low^ B-cells, and the CD19^+^CD21^low^/CD19^+^CD27^+^ ratio were all significantly elevated. A negative correlation was observed regarding the number of CD19^+^CD27^+^ memory B-cells (class-switched and non-class-switched), indicating both immunological reconstitution and association with resolution of cGVHD (data not shown).

**Table 5:** Parameters of immune reconstitution in patients with malignant underlying diseases: analyses of patients with resolved cGVHD compared to no cGVHD.

**Parameters**	**Resolved cGVHD**	**No cGVHD**	***P*-value**
Number of analyses	a = 65	a = 308	
Leukocytes × 10^3^ cells/ml	7,506.3	6,469.9	0.006
Monocytes × 10^3^ cells/ml	532.7	454.3	0.004
Granulocytes × 10^3^ cells/ml	4,687.3	3,738.6	0.001
IgG mg/dl	1,177.4	1,061.9	0.015
IgA mg/dl	157.4	131.7	0.023
IgE kU/L	235.1	126.6	0.035
IgG1 mg/dl	916.4	777.0	0.007
IgG3 mg/dl	73.9	57.3	0.004
CD19^+^CD27^+^IgD^−^ %	7.5	5.9	0.020
CD19^+^CD27^+^IgD^−^× 10^3^ cells/ml	29.9	22.3	0.018

*Mean values are depicted, and only significant values are shown*.

*a, number of analyses*.

As age and interval from HSCT significantly varied between the cGVHD and the no-cGVHD group, we performed a logistic regression analysis by additionally adjusting both parameters for the two groups: no significant influence on cellular and humoral markers was observed. Stepwise logistic regression for active cGVHD revealed a significant correlation between higher frequencies of CD19^+^CD21^low^ B-cells and the following parameters: younger age, high CD3^+^ and CD8^+^ T-cell numbers, lower CD4/CD8 ratio, lower CD19^+^ B-cells, and lower frequency of class-switched memory B-cells (data not shown).

### NIH-defined Severity of cGVHD Has an Impact on IR: Severe cGVHD Is Associated With Increased CD19^+^CD21^low^ B-cells

Finally, we examined whether parameters of IR would correlate with NIH-defined overall severity (mild, moderate, and severe) in several sub-analyses. Where analyses of the mild and moderate cGVHD group were compared with the severe cGVHD group, the latter showed a significant expansion of leukocytes (*p* = 0.007), monocytes (*p* = 0.013), granulocytes (*p* = 0.031), IgG4 (*p* = 0.014), CD3^+^ T-cells (*p* = 0.021), and CD8^+^ T-cells (*p* = 0.002). Again, the most severe disease manifestation correlated significantly with a distorted B-cell profile consisting of increased CD19^+^CD21^low^ B-cells (19.6% vs. 7.6%, *p* < 0.0001) along with an increased CD19^+^CD21^low^/CD19^+^CD27^+^ B-cell ratio (2.7% vs. 1.2%, *p* = 0.001, Table 6).

**Table 6:** Correlation of NIH-defined cGVHD overall severity (mild and moderate vs. severe) with parameters of immune reconstitution in patients with malignant underlying diseases.

**Parameters**	**Mild+moderate cGVHD**	**Severe cGVHD**	***P*-value**
Number of analyses	a = 42	a = 21	
Leukocytes × 10^3^ cells/ml	6,807.7	8,560.0	0.007
Monocytes × 10^3^ cells/ml	550.5	708.0	0.013
Granulocytes × 10^3^ cells/ml	4,301.0	5,400.0	0.031
IgG4 mg/dl	64.5	13.6	0.014
CD3^+^ × 10^3^ cells/ml	1,219.6	1,641.9	0.021
CD8^+^ × 10^3^ cells/ml	632.0	1,002.9	0.002
CD19^+^CD21^low^ %	7.6	19.6	0.001
Ratio CD19^+^CD21^low^/CD19^+^CD27^+^	1.2	2.7	0.001

*Mean values are depicted, and only significant values are shown*.

*a, number of analyses*.

**Figure 3:**
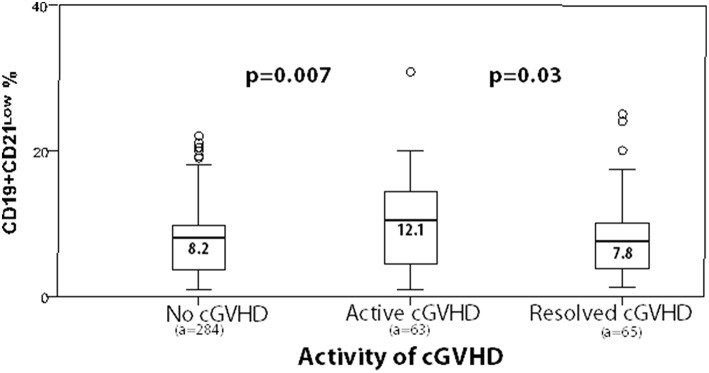
Box plots depict frequencies of CD19^+^CD21^low^ B-cells in correlation with activity of cGVHD at time of analyses (a, number of analyses).

**Figure 4:**
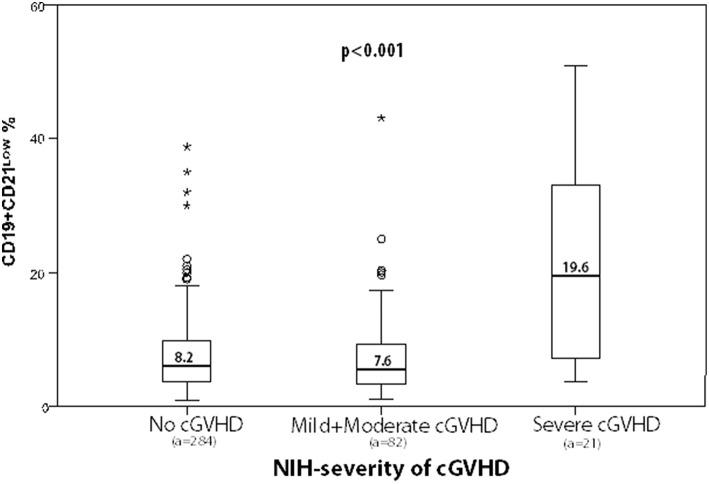
Box plots depict frequencies of CD19^+^CD21^low^ B-cells in correlation with NIH-defined overall severity of cGVHD (a, number of analyses). In SPSS, small circle identified an outlier whereas ^*^ is an extreme value.

We then correlated the expansion of CD19^+^CD21^low^ B-cells in all analyses with the activity and severity of cGVHD. Box plots depict the different characteristics of cGVHD according to CD19^+^CD21^low^ B-cells and show a significant expansion of CD19^+^CD21^low^ B-cells in association with the activity and severity of cGVHD (Figures 3, 4, Table 7). In fact, a mean of 19.6% of CD19^+^CD21^low^ B-cells was observed in the NIH severe group, while 7.6 and 8.2% were observed in the no-cGVHD and mild-to-moderate groups, respectively.

**Table 7:** Chronic GVHD characteristics of patients with malignant underlying diseases.

	***n* = 29 (%)**
Median time from HSCT to onset of cGVHD in months (range)	6 (3–18)
Median duration of cGVHD at time of analysis (median, range) in months	20 (2–208)
**Acute GVHD**	26 (90)
Grades III–IV	8 (31)
Grades II–IV	15 (58)
**Onset type of cGVHD**	
Progressive	9 (31)
Quiescent	17 (59)
*De novo*	3 (10)
**NIH classification at onset**	
Classic chronic	18 (62)
Overlap	11 (38)
**Overall severity grading of cGVHD at maximum**	
Mild	4 (14)
Moderate	6 (21)
Severe	19 (65)
**Organ involvement at maximum**	
Skin	26 (90)
Oral mucosa	25 (86)
Eyes	13 (45)
Joints	11 (38)
Gastrointestinal	7 (24)
Liver	9 (31)
Genital	5 (17)
Lung	5 (17)

### Sensitivity and Specificity of Biomarkers in cGVHD

Subsequently, we evaluated the above identified parameters pointing toward cGVHD activity by receiver operating characteristic analysis to identify a cutoff value and the sensitivity that corresponded to a >80% specificity in the malignant disease group. We determined a conservative cutoff value for each parameter with what should be considered to be positively correlated with activity of cGVHD for the following: percentages of CD19^+^CD21^low^ B-cells (>11.49%), CD56^+^ NK cells (>360 × 10^3^/ml), IgM (>168 G/L), CD19^+^CD21^low^/CD19^+^CD27^+^ B-cell ratio (>1.32), monocytes (>600 × 10^3^/ml), and CD19^+^CD27^+^ IgD^−^ class-switched memory B-cells (≤7.76%) at 80% specificity (Table 8).

**Table 8:** ROC curve analysis for patients with malignant diseases.

**Parameter**	**Cutoff within 80% specificity**	***P*-value**	**AUC**	**95% confidence interval**
CD19^+^CD21^low^ %	11.49	0.04	0.581	0.529–0.632
CD56^+^ × 10^3^ cells/ml	>360	0.062	0.576	0.522–0.629
IgM mg/dl	>168	0.62	0.522	0.466–0.578
CD19^+^CD27^+^IgD^−^× 10^3^ cells/ml	≤ 7.76	0.0002	0.643	0.581–0.702
Monocytes × 10^3^ cells/ml	>600	0.0045	0.624	0.570–0.676
Ratio CD19^+^CD21^low^/CD19^+^CD27^+^	>1.32	<0.0001	0.671	0.609–0.729

*AUC, area under the curve; ROC, receiver operating characteristic*.

## Discussion

Here, we have analyzed whether cellular and humoral parameters of IR in 146 children during long-term FU of a median of 8.6 years including 659 individual analyses would correlate with the stratification of NIH-defined cGVHD. We here show for the first time in a pediatric cohort that both cGVHD and its activity during the disease course are associated with the perturbation of the B-cell compartment, including the previously described circulating CD19^+^CD21^low^ B-cells. Our findings are of importance since failure to properly immune-reconstitute after HSCT may lead to complications such as GVHD, infections, relapses, or secondary malignancies (5–7), and published data on pediatric IR are limited, i.e., lacking validated markers for pediatric cGVHD. Moreover, immune monitoring parameters are difficult to harmonize due to the considerable variety of underlying diseases (malignant and non-malignant), the generally lower incidence of GVHD, and thus the limited number of comparable patients within study collectives (30). Herein, a long-term observational period was chosen on purpose to (i) reduce the influence of engraftment kinetics and (ii) enhance the number of evaluations of individual cases with cGVHD activity (2, 31).

Data about IR, cGVHD, and possible biomarkers often combine adult and pediatric patients and fail to differentiate between the underlying diseases (3, 7, 17). We observed significant disparities concerning (i) age, which may influence the kinetics of IR (32, 33), and (ii) transplant characteristics, like the use of mismatched donors, PBSCs, TBI, TCD, and ATG between the malignant and the non-malignant cohort. The use of TBI, mismatched donors, and PBSCs are known risk factors for aGVHD (29), consistent with the significantly higher incidence of aGVHD we observed in the malignant disease group (Table 1). As aGVHD is the main risk factor for subsequent cGVHD, these parameters are crucial when studying the impact of cGVHD. Moreover, IR may be affected by the stem cell source used and the application of ATG (9). In accordance, we observed a higher incidence of cGVHD occurring at a significantly shorter interval from HSCT (30% vs. 18%; median, 6 months vs. 10 months, respectively) for the malignant cohort. Therefore, it remains questionable if parameters of cGVHD-related immune dysfunction are comparable between malignant and non-malignant pediatric HSCT patients.

In our study, we initially monitored IR dynamics in the absence of the immunopathology/dysregulation caused by cGVHD during long-term FU. The comparison between early and late FU data demonstrated the efforts of the newly established immune system to reconstitute the host, similar to studies by D'Orsogna et al. (5) and van den Brink et al. (34) investigating adult collectives after HSCT and children (9), recently (35, 36). Although, overall CD19^+^ B-cell numbers normalized within the first 365 days post-transplant, dissection of the B-cell compartment showed a protracted reconstitution with low proportions of memory B-cells and a high degree of naivety/immaturity due to the constant recruitment of B-lymphocytes from the BM, which is a typical feature of the newly establishing immune system (9, 37). Concomitantly, antibody production deficiency, with low IgG and IgA levels, was evident during early FU. The maturation block of IgM memory B-cells contributed to impaired humoral IR in children early after HSCT as demonstrated recently (38). In addition, even in the absence of cGVHD, we found CD19^+^CD21^low^ B-cells significantly elevated until day +365 when compared to late FU.

Subsequently, we analyzed the correlation between cGVHD and the parameters of IR. Here, we observed a significant elevation of distinct parameters such as leukocytes, monocytes, granulocytes, and NK cells (Table 3). Elevated NK cells are an early prognostic factor for GVHD development, while prolonged NK cell expansions have been described to be associated with chronicity of GVHD (39). The observed monocytosis highlights the importance of further studying the etiology of cGVHD, which might be related to chronic inflammation, as observed in autoimmune processes (40) or functional asplenia (41). Macrophage colony-stimulating factor (M-CSF) is a well-known inducer of monocytosis, and its administration to patients undergoing HSCT was shown to attenuate cGVHD (42), implicating that also, after HSCT, elevated M-CSF levels might drive monocyte expansion and alleviate cGVHD. Higher absolute monocyte counts were related with cGVHD, future cGVHD onset, higher NRM rate, as well as poor outcomes of allogeneic HSCT (43), and *vice versa*, monocyte recovery during the first year after HSCT may be associated with better outcome (44).

In addition to the innate, the adaptive immune system also was compromised by the occurrence of cGVHD in patients included in the current study. In particular, active cGVHD correlated with a low proportion of CD19^+^CD27^+^ memory B-cells, confirming memory B-cell deficiency as a risk factor/marker for persistence of cGVHD (8, 15, 45). Although we observed that CD19^+^CD27^+^ memory B-cells tended to increase during long-term FU, the increase was significantly lower in the active when compared to both the resolved cGVHD and the control group (Tables 3, 4). This may identify continuously low numbers of memory B-cells as a predictive marker for developing cGVHD during later FU in pediatric HSCT patients. The association of cGVHD with perturbed B-cell homeostasis has been shown in a number of studies performed previously (25, 46). In fact, the correlation between severity and activity of cGVHD has been clearly demonstrated (15, 25), with CD19^+^CD21^low^ B-cells identified as a marker in a prospective study (15). While in children, the clinical relevance of certain B-cell subsets (CD19^+^CD21^low^ B-cells) has been proposed in patients with common variable immunodeficiency (CVID) (47), no such data were available for pediatric HSCT patients.

Whether the observed accumulation of CD19^+^CD21^low/neg^ B-cells in the collective of cGVHD patients is primarily due to persistent activation/inflammation within the cGVHD milieu or, alternatively, the consequence of CD19^+^CD21^low/neg^ B-cells being causally involved in disease pathogenesis remains an unresolved issue (15, 27). An elevation of exhausted CD19^+^CD21^−^CD27^−^CD10^−^ B-cells in active cGVHD was described recently (48), implicating these cells as a potential biomarker for severity of cGVHD (48). Enlargement of the CD19^+^CD21^low^CD27^−^ tissue-like memory B-cell pool along with features of B-cell exhaustion and reduced BCR-induced Ig-secreting capacity was also demonstrated in individuals with hepatitis C infection (20), Sjogren's syndrome (21, 22), and HIV (23, 24).

Moreover, expanded CD19^+^CD21^low^CD38^low^ B-cell subsets were observed in CVID (49, 50), SLE (51), and rheumatoid arthritis (52). These innate-like B-cells were refractory to antigenic stimulation via their BCR and contained broadly autoreactive, tissue homing clones (17, 52–54). It is well-accepted that chronic hyperactivation is generating a milieu advantageous for breaking B-cell tolerance and inhibiting negative selection and maturation of B-cells (17–19). A pathophysiologic link between the abundance of IFN-producing CD4^+^ T follicular helper cells and the appearance of CD19^+^CD21^low^ B-cells was made in CVID patients recently (55), which might be linked to the overexpression of spleen tyrosine kinase (56). However, increased C3d generation and the formation of immune complexes might similarly account for downregulation of the CD21 molecule associated with altered/autoagressive B-cell function (57). The elevation of IgM and IgG1–3 subclasses observed herein in the active cGVHD study group might result from such autoreactive B-cells, which are frequently detected in GVHD (58–60).

Importantly, the resolution of cGVHD significantly correlated with the expansion of CD19^+^CD27^+^ memory B-cells, which was accompanied by the normalization of CD19^+^CD21^low^ B-cell frequencies, involving both non-class-switched and class-switched memory B-cells, resulting in a significant decrease of the CD19^+^CD21^low^/CD19^+^CD27^+^ ratio. While the proportions of CD19^+^CD21^low^ B-cells significantly decreased in the no-cGVHD group and normalized in the resolved cGVHD cohort, they remained significantly higher (cutoff within 80% of specificity >11.49%) in the active cGVHD group (Table 4; Figure 2). IgG and IgE levels were significantly (pathologically) elevated in resolved cGVHD (61). Elevated IgE levels have been observed in patients with aGVHD and solid allograft rejection previously (62, 63), the latter cohort mounting functionally relevant HLA-specific IgE (64). These findings are in contrast to other studies, including our study, implying elevated serum IgE as marker for robust, post-transplant IR (65).

Our study has certain limitations because features of exhaustion, expression of chemokine/adhesion molecules determined in detail, and functional analyses could not be conducted, due to limited biological material from patients. As outlined by cGVHD expert groups elsewhere, details of immunosuppressive treatment would be important in the context of studies on IR and are an unmet need.

In summary, we here report on the significant association of the activity and the severity of NIH-defined cGVHD with low CD19^+^CD27^+^ B-cells and the expansion of CD19^+^CD21^low^ B-cells in a well-defined pediatric HSCT cohort. Likewise, we show other significant but, compared to adult data, different disturbances of IR, such as early reconstitution of circulating CD19^+^ B-cells without the influence of cGVHD, and a significant elevation of leukocytes, monocytes, granulocytes, NK cells, and both CD3^+^ and CD8^+^ T-cells but not CD4^+^ T-cells in cGVHD patients. Our data also provide evidence that the interval from HSCT as well as cGVHD activity may be of critical importance for the detailed investigation of pediatric cohorts (9, 13). Finally, we prove that the differences in risk factors and patterns of IR between malignant and non-malignant diseases are important for identifying cGVHD biomarkers.

## Data Availability

The raw data supporting the conclusions of this manuscript will be made available by the authors, without undue reservation, to any qualified researcher.

## Ethics Statement

Ethics approval for this project in accordance with the Declaration of Helsinki and the institutional review board of the Ethics Committee from the Medical University of Vienna and the St. Anna Children's Hospital had been obtained.

## Author Contributions

AL designed the study. AL and ZK analyzed the data and wrote the manuscript. WP performed flow cytometric analyses. HG and WP critically revised the manuscript. EG, AJ, and DB collected the data. UK, AR, and GF performed FACS analyses. CP revised the manuscript. All authors approved the final version of the manuscript to be submitted.

### Conflict of Interest Statement

HG served in advisory boards and received speaker's fees for participation in scientific meetings of the companies Therakos, Novartis, and Roche. The remaining authors declare that the research was conducted in the absence of any commercial or financial relationships that could be construed as a potential conflict of interest. The reviewer M-TL-S and handling editor declared their shared affiliation.

## Abbreviations

ATG: antithymocyte globulin
AUC: area under the curve
BCR: B-cell receptor
BM: bone marrow
CI: confidence interval
CsA: cyclosporine
CVID: common variable immunodeficiency
FK506: tacrolimus
HSCT: allogeneic hematopoietic stem cell transplantation
HLA: human leukocyte antigen
HIV: human immunodeficiency virus
GVHD: graft-vs.-host disease
MTX: methotrexate
MMF: mycophenolate mofetil
IR: immune reconstitution
Ig: immunoglobulin levels
IFN: interferon
FU: follow-up
M-CSF: macrophage colony-stimulating factor
MAC: myeloablative conditioning
MRD: matched related donor
MUD: matched unrelated donor
MMRD: mismatched related donor
MMUD: mismatched unrelated donor
NIH: National Institutes of Health
NK cell: natural killer cell
NRM: non-relapse mortality
RIC: reduced-intensity conditioning
ROC: receiver operating characteristic
PB: peripheral blood
PBSC: peripheral blood stem cells
SLE: systemic lupus erythematosus
VNTR: variable number tandem repeat
TBI: total body irradiation
TCD: T-cell depletion.
