# Supplementary material for: National Institutes of Health–Defined Chronic Graft-vs.-Host Disease in Pediatric Hematopoietic Stem Cell Transplantation Patients Correlates With Parameters of Long-Term Immune Reconstitution
Source: Front Immunol. 2019 Aug 27;10:1879. doi: 10.3389/fimmu.2019.01879 (PMC6718560; doi:10.3389/fimmu.2019.01879)
Supplement: Supplementary file 1 [file Table_1.DOCX]

Supplementary Material

Supplemental Table 1. General patient characteristics

| **Number of patients**  **n=139** | **cGVHD**  **n=38 (26%)** | **No cGVHD**  **n=101 (69%)** | **p**  **(x² test)** |
| --- | --- | --- | --- |
| **Number of patients**  **Median age at HSCT in years (range)** | 10  (0.5-23.8) | 8,6  (0.1-23.4) | n.s. |
| **Male** | 21 (55) | 71 (70) | n.s. |
| **Female** | 17 (45) | 30 (30) |  |
| ***Diagnosis*** |  |  |  |
| **Malignant**  **Lymphoid**  **Myeloid**  **Solid tu, sec. malign.** | 29 (76)  16 (55)  13 (45)  0 | 62 (61)  35 (56)  24 (39)  3 (5) | n.s. |
| **Non-malignant**  **Immunodeficiency**  **Hb-pathies**  **BM-failure**  **Metabolic**  **Phagocytic**  **Autoimmune** | 9 (24)  2 (22)  1 (11)  1 (11)  2 (22)  2 (22)  1 (11) | 39 (39)  15 (38)  5 (13)  9 (23)  3 ( 8)  7 (18)  0 |  |
| ***Conditioning regimen*** |  |  |  |
| **MAC** | 25 (66) | 54 (53) | n.s. |
| **RIC** | 13 (34) | 47 (47) |  |
| **TBI containing** | 20 (53) | 40 (40) | n.s. |
| **TBI+MAC** | 17 (85) | 38 (95) | n.s. |
| **TCD** | 4 (11) | 17 (17) | n.s. |
| ***Stem cell donors*** |  |  |  |
| **MRD**  **MUD**  **MMRD**  **MMUD** | 14 (37)  22 (58)  2 ( 5)  0 | 38 (38)  51 (51)  9 ( 9)  3 ( 3) | n.s. |
| ***Stem cell source*** |  |  |  |
| **Bone marrow** | 27 (71) | 72 (71) | n.s. |
| **PBSCs** | 11 (29) | 29 (29) |  |
| **Median number of CD34^+^ cells x10^6^/kg (range)** | 6.1  (0.9-62) | 5.7  (0.24-32.5) | n.s. |
| ***GVHD prophylaxis*** | | | |
| **No GVHD prophylaxis** | 1 (3) | 4 (4) | n.s. |
| **Cyclosporine only** | 13 (34) | 36 (35) | n.s. |
| **Cyclosporine + MTX** | 12 (32) | 32 (32) | n.s. |
| **Cyclosporine + MMF** | 10 (24) | 27 (26) | n.s. |
| **CSA+MTX+MMF** | 2 ( 5) | 2 ( 2) | n.s. |
| **+ ATG *** | 22 (59) | 77 (76) | 0.0381 |
| ***Acute GVHD*** | 33 (87) | 53 (53) | 0.0002 |
| **Grade 0** | 5 (13) | 48 (48) |  |
| **Grade 0-I** | 19 (50) | 87 (86) | 0.0001 |
| **Grades I-II** | 23 (70) | 47 (89) | 0.0444 |
| **Grades III-IV** | 10 (30) | 6 (11) |  |
| **Grade II-IV** | 19 (58) | 14 (26) | 0.0060 |

Abbreviations: Cyclosporine (CSA), methotrexate (MTX ), mycophenolic acid (MMF), * anti-thymocyte globulin (ATG) was given additionally to other conditioning medications. Reduced intensity conditioning (RIC), total body irradiation, (TBI) , number of patients (n)

Hematopoietic stem cell transplantation (HSCT), Hemaglobinopathies (Hb-pathies), bone marrow (BM), myeloablative conditioning (MAC), T cell depletion (TCD), matched related donor (MRD), matched unrelated donor (MUD), mismatched related donor (MMRD), mismatched unrelated donor (MMUD), Peripheral Blood Stem cells(PBSCs)

Supplemental Table 2. Age at time point of analyses and interval from HSCT to analyses

| **Number of analyses** | **All**  **a=659*** | **Classic cGVHD a=187** | **No cGVHD**  **a=443** | **p**  **(x² test)** |
| --- | --- | --- | --- | --- |
| **Median age at time of analysis in years (range)** | 12.4  (0.6-32.9) | 13.4  (1.6-32.9) | 12  (0.6-26.5) |  |
| **<8 years** | 168/659 (25%) | 31/187 (17%) | 134/443 (30%) | 0.0003 |
| **≥8 years** | 491/659 (75%) | 156/187 (83%) | 309/443 (70%) |  |
| **Interval between HSCT and analysis in months** | 37  (3-190) | 54  (3-190) | 29  (3-186) |  |
| **≤12 months** | 125/659 (19%) | 23/187 (12%) | 96/443 (22%) | 0.0054 |
| **>12 months** | 534/659 (81%) | 164/187 (88%) | 347/443 (78%) |  |

- Excluding late acute GVHD, a – number of analyses

Supplemental Table 3. Summary of directly conjugated monoclonal antibodies (mAbs)

Following antibodies were used: mAbs CD3-APC (S4.1), CD16-PE (3G8), CD19-FITC (SJ25-C1), CD21-PE (BU32), CD56-PE (MEM-188), negative control IgG1-APC, and goat affinity purified F(ab‘)2 anti-human IgD antibody from Caltag Laboratories (Burlingame, CA), CD4-FITC (VIT4), CD8-PE (VIT8), CD14-PE (MEM-18), CD45-FITC (VIT200), and negative control mAbs VIAP-FITC and 4H1-A7-PE from An der Grub (Kaumberg, Austria) and CD69-FITC mAb (L78) from Becton Dickinson (San Jose, CA). CD27-FITC (VIT14)

Supplemental Table 4. Immunoglobulin levels adjusted for age according to the reference lab

| **Immunoglobulin** | **IgG** | **IgM** | **IgA** | **IgE** |
| --- | --- | --- | --- | --- |
| Unit | mg/dl | mg/dl | mg/dl | kU/L |
| <1mo | 700-1600 | 10-30 | 7-94 | <1.5 |
| 1-12mo | 250-750 | 10-70 | 10-131 | <15 |
| 1-3 years | 180-800 | 20-100 | 19-220 | <60 |
| 3-5y | 300-1000 | 30-100 | 48-345 | <90 |
| 5-7y | 350-1000 | 40-140 | 41-297 | <90 |
| 7-10y | 500-1300 | 40-180 | 51-297 | <90 |
| 10-13y | 700-1400 | 40-150 | 44-395 | <200 |
| >13y | 700-1600 | 40-230 | 70-400 | <100 |

| Age | **IgG1** | **IgG2** | **IgG3** | **IgG4** |
| --- | --- | --- | --- | --- |
| Units | mg/dl | mg/dl | mg/dl | mg/dl |
| <12mo | 140-620 | 41-130 | 11-85 | 0-0.8 |
| 12-24mo | 170-720 | 40-180 | 12-91 | 0-40.8 |
| 2-3y | 240-780 | 55-200 | 15-93 | 0.6-68.9 |
| 3-4y | 270-810 | 65-220 | 16-96 | 1.2-93.8 |
| 4-6y | 300-840 | 70-255 | 17-97 | 1.7-115.7 |
| 6-9y | 350-910 | 85-330 | 20-104 | 3-157.7 |
| 9-12y | 370-930 | 100-400 | 22-109 | 4.3-190 |
| 12-18y | 370-910 | 110-485 | 24-116 | 5.2-196 |
| >16y | 280-800 | 115-570 | 24-125 | 5.2-125 |
